# Ezetimibe use and mortality after myocardial infarction: A nationwide cohort study^☆^

**DOI:** 10.1016/j.ajpc.2024.100702

**Published:** 2024-06-23

**Authors:** Ville Kytö, Aleksi Tornio

**Affiliations:** a Heart Center Turku University Hospital and University of Turku, Turku, Finland; b Turku Clinical Research Center, Turku University Hospital, Turku, Finland; c Integrative Physiology and Pharmacology, Institute of Biomedicine, University of Turku, Turku, Finland; d Unit of Clinical Pharmacology, Turku University Hospital, Turku, Finland

**Keywords:** Myocardial infarction, Ezetimibe, Cholesterol, Outcome

## Abstract

**Background:**

The inhibition of intestinal cholesterol absorption by ezetimibe improves outcomes after myocardial infarction (MI), yet real-world data on ezetimibe is scarce. We studied the usage of ezetimibe and association with outcome after MI.

**Methods:**

Consecutive MI patients in Finland (2010–2018) were retrospectively studied (*N* = 57,505; 65 % men; mean age 69 years). The study data were collected from national registries. The median follow-up was 4.5 (IQR 2.8–7.1) years. Between-group differences were adjusted for using multivariable regression. Ezetimibe use was studied with competing risk analyses.

**Results:**

The cumulative incidence of ezetimibe use was 3.7 % at 90 days, 13.4 % at 5 years, and 19.8 % at 10 years. Younger age was one of the strongest predictors of ezetimibe use (adj.sHR 6.67; CI 5.88–7.69 for patients aged <60 vs ≥80 years). Women were more likely to use ezetimibe during follow-up than men. The average proportion of patients using ezetimibe during follow-up was 6.8 %. (11.7 % at 10 years). Ezetimibe was discontinued by 43.6 % of patients during follow-up. Patients with early ezetimibe therapy after MI had lower all-cause mortality during follow-up (33.6% vs 45.1 %; adj.HR 0.77; CI 0.69–0.86; *P* < 0.0001). Early ezetimibe use was associated with lower mortality irrespective of sex, age, atrial fibrillation, diabetes, heart failure, malignancy, revascularization, or statin use. Ongoing ezetimibe therapy during follow-up was associated with lower mortality in a time-dependent analysis (adj.HR 0.53; CI 0.48–0.59; *P* < 0.0001).

**Conclusions:**

Ezetimibe is associated with a lower risk of death after MI, yet its therapeutic use is limited, and discontinuation is frequent.

## Graphical abstract

**Figure:**
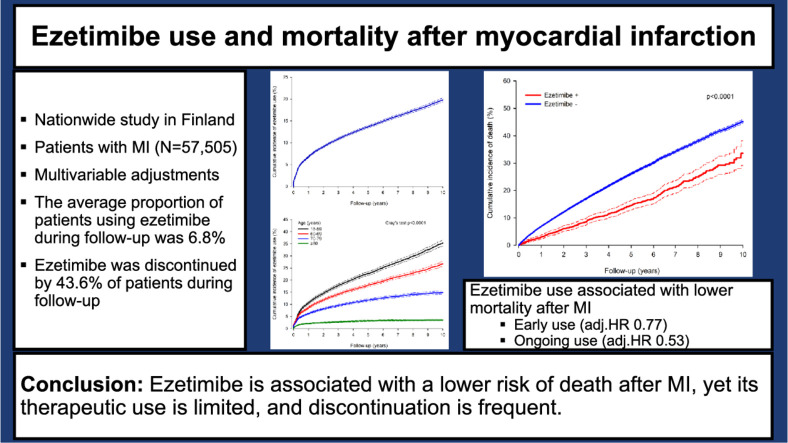

## 1 Introduction

Lowering low-density lipoprotein (LDL) cholesterol with effective pharmacotherapy plays a key role in secondary prevention after myocardial infarction (MI) [[1], [2], [3]]. Statins are the first-line mainstay therapy after ischemic events [1], yet their usage and intensity are commonly suboptimal [4]. Ezetimibe is readily available, non-statin drug that lowers LDL cholesterol levels by inhibition of intestinal cholesterol [5]. The Improved Reduction of Outcomes: Vytorin Efficacy International Trial (IMPROVE-IT) showed the benefit of ezetimibe in reducing cardiovascular events after acute coronary syndrome (ACS) when combined with statins [6]. Ezetimibe is well tolerated and has a safety profile comparable to placebo when added to statin [6].

Ezetimibe is recommended by lipid guidelines for secondary prevention after ACS when the LDL levels remain elevated with maximum tolerated statin therapy [1,2]. However, observational real-world data on ezetimibe after MI is limited. Thus, we studied the usage of ezetimibe and association with outcome after MI in a real-life nationwide setting.

## 2 Methods

### 2.1 Study population

We studied consecutive patient with incident MI in Finland between Jan 1st, 2010 to Dec 31st, 2018. Patients surviving 90 days after MI discharge were retrospectively identified from the Care Register for Healthcare in Finland (CRHF), a nation-wide mandated-by-law database including data on all hospital admissions and major interventional procedures in Finland [7]. All hospitals treating patients with MI (*N* = 20, of which five have emergency cardiac surgery available) were included in the study. Patients with missing follow-up data (*N* = 352, 0.6 %) were excluded.

### 2.2 Outcomes and definitions

The outcomes of interest were ezetimibe use and all-cause death. Drug usage was defined by records of drug purchase from a pharmacy. In Finland, ezetimibe is only available from pharmacies with a prescription, and it is dispensed for a maximum of three-month usage at a time [8]. All purchases are recorded in the national database used in the study. Ezetimibe was detected using ATC codes C10AX09, C10BA02, C10BA05, and C10BA06. The MI index was identified with ICD-10 code I21 as the primary discharge diagnosis. Co-morbidities, MI type, and revascularization were detected using a combination of national registries as previously defined (Supplemental Methods) [9,10]. Early ezetimibe use was defined as use within 90 days after MI. Usage of cardiovascular medications and statin therapy intensity within 90 days after MI were also detected [11]. Ongoing ezetimibe use and discontinued use were studied (Supplemental Methods). In addition, associations of baseline characteristics of patients using early ezetimibe with 10-year mortality were studied. The follow-up was 10 years and continued up to Dec 31, 2020. The median follow-up period was 4.5 (IQR 2.8–7.1; max 10) years.

### 2.3 Data sources and permissions

The data were obtained from Findata and the National Institute for Health and Welfare of Finland (CRHF, Finnish cancer registry, medication purchases; permission THL/164/14.02.00/2021) and Statistics Finland (mortality; permission TK-53–484–20). The used registries are mandatory by law and offer full coverage of the Finnish population [12]. The requirement for informed consent was waived by law due to the study design. The participants were not contacted. The legal basis for the processing of personal data was public interest and scientific research (EU General Data Protection Regulation 2016/679 [GDPR], Article 6(1)(e) and Article 9(2)(j); Data Protection Act, Sections 4 and 6).

### 2.4 Statistical analysis

The outcomes were studied using the Kaplan-Meier estimator and Cox regression (death) or the cumulative incidence function and Fine-Gray regression accounting for the competing risk of death (ezetimibe use) [13]. Multivariable analyses were adjusted with the following patient characteristics: baseline age, sex, atrial fibrillation, chronic pulmonary disease, cerebrovascular disease, dementia, diabetes, heart failure, hypertension, liver disease, malignancy, peripheral vascular disease, prior myocardial infarction, psychotic disorder, rheumatic disease, renal failure, revascularization, MI type, intensity of initial statin dose, usage of ACEi/ARB, aldosterone antagonist, antiarrhythmic medication, beta-blocker, digoxin, oral anticoagulation, or P2Y12 inhibitor therapy. Subgroup analyses were performed in patients grouped by sex, age (18–59, 60–69, 70–79, and ≥ 80 years), atrial fibrillation, diabetes, heart failure, malignancy, revascularization, and statin therapy after MI using interaction analyses. The association of ongoing ezetimibe with the primary outcome was studied using time-dependent Cox regression [14]. Potential impact of residual confounding was estimated by the E-value [15].

The results were given as the mean, median, percentage, hazard ratio (HR), or sub-distribution HR (sHR) with a 95 % confidence interval (CI), IQR, or ± standard deviation (SD). Statistical significance was inferred at *P* < 0.05. SAS version 9.4 (SAS Institute Inc., Cary, NC, USA) was used for the analyses.

## 3 Results

The study included 57,505 patients (mean age 69.3 years, SD 12.5; 65.1 % male), of whom 3.7 % used ezetimibe within 90 days after MI. Baseline features, treatments, co-morbidities, and other pharmacotherapies used early after MI are presented in Table 1. Patients with early ezetimibe use were younger, more frequently revascularized, had more commonly diabetes, hypertension, or established vascular disease, and used ACEi/ARBs, beta-blockers, or P2Y12 inhibitors more commonly than patients without ezetimibe (Table 1).

**Table 1:** Baseline features of patients by early usage of ezetimibe after myocardial infarction.

Variable	All patients *N* = 57,507	Early ezetimibe *n* = 2105	No early ezetimibe *n* = 55,402	Between group *P* value
Age, mean (SD) years	69.3 (12.5)	65.1 (11.0)	69.5 (12.5)	<0.0001
Female	34.9 %	31.9 %	35.0 %	0.003
Medical history				
Atrial fibrillation	15.5 %	12.9 %	15.6 %	0.001
Cerebrovascular disease	12.8 %	15.0 %	12.7 %	0.002
Chronic pulmonary disease	13.9 %	14.4 %	13.9 %	<0.0001
Dementia	4.9 %	1.4 %	5.1 %	<0.0001
Depression	10.5 %	10.0 %	10.6 %	0.440
Diabetes	26.7 %	33.2 %	26.5 %	<0.0001
Insulin dependent	8.9 %	11.5 %	8.8 %	<0.0001
Non-insulin dependent	17.8 %	21.7 %	17.7 %	<0.0001
Heart failure	18.7 %	15.3 %	18.9 %	<0.0001
Hypertension	53.2 %	57.6 %	53.1 %	<0.0001
Liver disease	3.7 %	3.9 %	3.7 %	0.686
Malignancy	14.3 %	12.0 %	14.3 %	0.003
Peripheral vascular disease	8.5 %	12.5 %	8.3 %	<0.0001
Prior myocardial infarction	13.9 %	19.8 %	13.7 %	<0.0001
Psychotic disorder	3.2 %	2.0 %	3.3 %	0.001
Rheumatic disease	6.6 %	6.8 %	6.6 %	0.678
Renal failure	3.7 %	4.5 %	3.7 %	0.053
Revascularization	66.2 %	75.1 %	65.9 %	<0.0001
PCI	59.5 %	68.5 %	59.2 %	<0.0001
CABG	7.5 %	7.4 %	7.5 %	0.780
ST-elevation MI	36.8 %	33.2 %	37.0 %	0.001
Ezetimibe prior to MI	1.2 %	24.4 %	0.3 %	<0.0001
Statins after MI	85.3 %	86.0 %	85.2 %	<0.0001
Statin intensity				<0.0001
High	38.4 %	51.5 %	37.9 %	
Moderate	58.6 %	44.1 %	59.2 %	
Low	3.0 %	4.4 %	2.9 %	
Other pharmacotherapy after MI				
ACEi or ARB	70.8 %	75.1 %	70.6 %	<0.0001
Aldosterone antagonist	4.2 %	4.1 %	4.2 %	0.730
Antiarrhythmic	1.2 %	1.0 %	1.2 %	0.302
Beta-blocker	83.1 %	84.8 %	83.1 %	0.038
Digoxin	2.0 %	1.3 %	2.0 %	0.024
Oral anticoagulation	15.6 %	14.3 %	15.6 %	0.091
P2Y_12_ inhibitor	74.0 %	84.9 %	74.6 %	<0.0001
PCSK9 inhibitor	0.003 %	–	0.004 %	<0.0001

Abbreviations: PCI, percutaneous coronary intervention; CABG, coronary artery bypass grafting; MI, myocardial infarction.

### 3.1 Ezetimibe usage

**Fig. 1:**
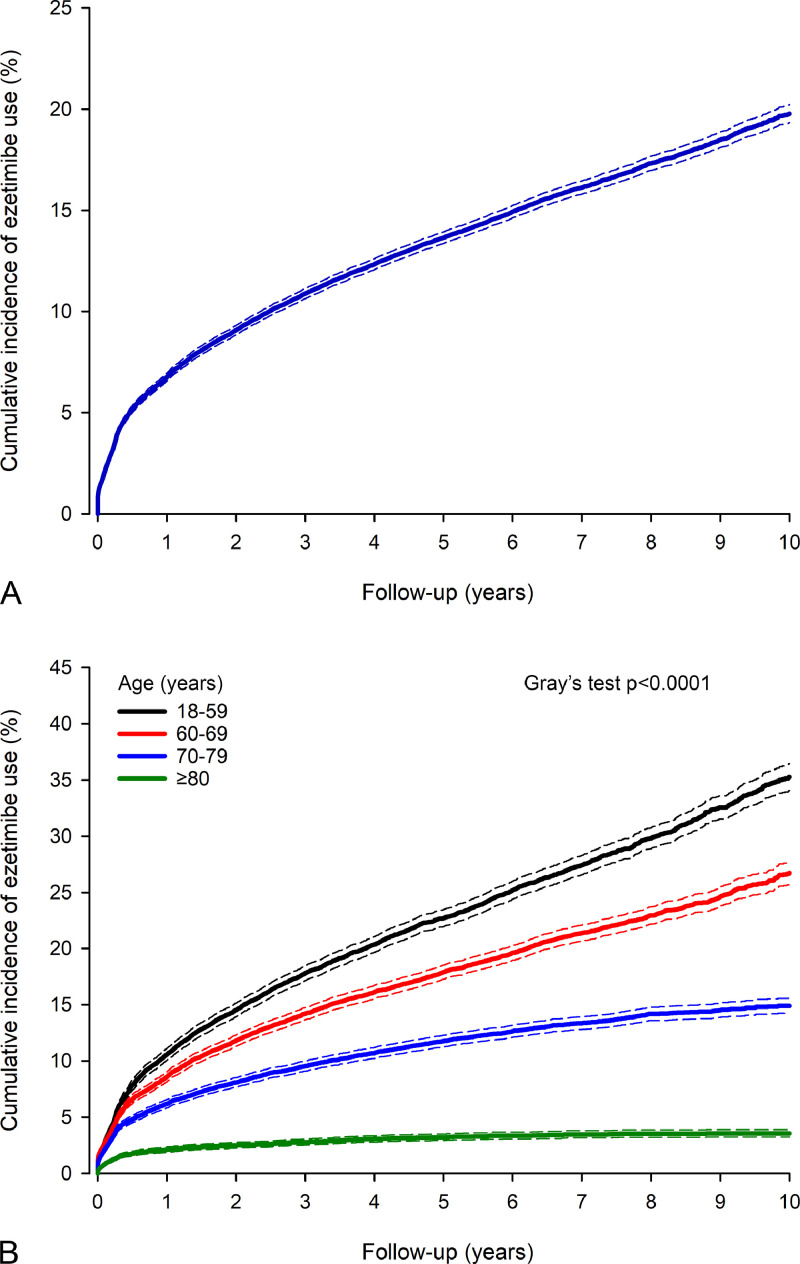
Cumulative incidence of ezetimibe usage after myocardial infarction in all patients (A) and by age (B). Competing risk analyses. Dashed lines represent 95 % confidence intervals. Please note the difference in the y-axis.

**Fig. 2:**
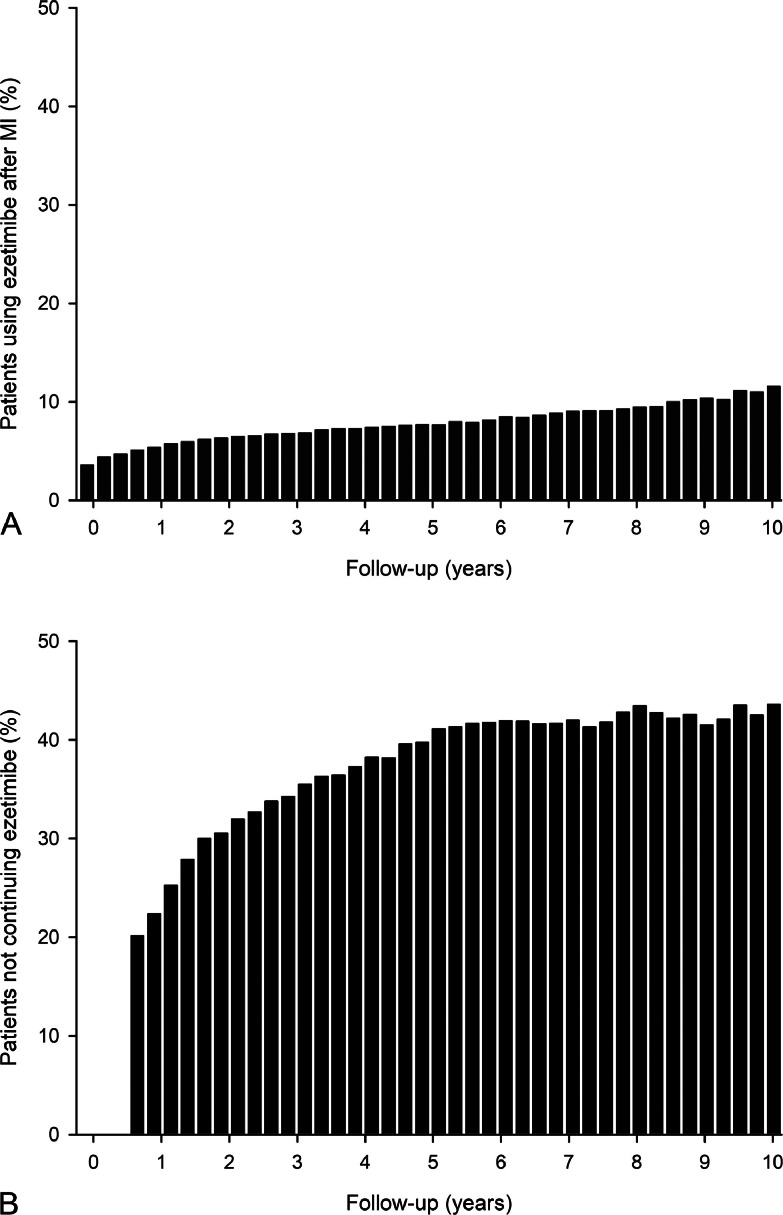
Proportions of patients using ezetimibe (A) and patients not continuing ezetimibe (B) during follow-up after myocardial infarction.

The cumulative incidence of ezetimibe use was 6.8 % at 1 year, 13.4 % at 5 years, and 19.8 % at 10 years of follow-up (Fig. 1). Therapy initiation occurred most actively during the first year after MI (Fig. 2). Younger age was one of the strongest predictors of ezetimibe usage after MI in the unadjusted and adjusted analysis (Table 1). The cumulative incidence for ezetimibe therapy was 35.3 % in patients aged < 60 years, 26.7 % in patients aged 60–69, 14.9 % in patients aged 70–69, and 3.5 % in patients aged ≥ 80 years (*P* < 0.0001) at 10 years after MI (Fig. 2). The slope of the cumulative incidence was notably steeper in the younger MI patients, with increasing age difference during follow-up (Fig. 2).

Women were more likely to initiate ezetimibe therapy after MI when accounting for age, comorbidities, and other co-variables (adj.sHR 1.23; CI 1.18–1.31; *P* < 0.0001). Hypertension, peripheral vascular disease, and having a history of previous MI were associated with a higher likelihood of ezetimibe use in the multivariable analysis. Dementia, depression, diabetes, heart failure, and psychotic disorder were independently associated with a lower likelihood of ezetimibe use in the long term (Table 2). Revascularized patients were treated with ezetimibe more frequently (Table 2). Patients not using early statins were less likely to receive ezetimibe in the long term. High-intensity statin dosing early after MI was associated with ezetimibe use during follow-up (Table 2).

**Table 2:** Association of patient baseline features with ezetimibe usage after myocardial infarction in 10-Year Follow-up. Results of competing risk univariable and multivariable regression models.

Variable	Univariable		Multivariable	
	sHR (95 %CI)	*P* Value	sHR (95 %CI)	*P* Value
Age (years)		<0.0001		<0.0001
≥ 80	Reference	Reference	Reference	Reference
70–79	4.04 (3.65–4.46)	<0.0001	3.24 (2.92–3.59)	<0.0001
60–69	6.76 (6.14–7.44)	<0.0001	5.10 (5.60–5.65)	<0.0001
18–59	9.08 (8.25–9.99)	<0.0001	6.67 (5.88–7.69)	<0.0001
Female sex	0.77 (0.73–0.80)	<0.0001	1.23 (1.17–1.29)	<0.0001
Medical history				
Atrial fibrillation	0.52 (0.49–0.56)	<0.0001	0.95 (0.88–1.02)	0.157
Cerebrovascular disease	0.66 (0.61–0.71)	<0.0001	1.02 (0.95–1.10)	0.613
Chronic pulmonary disease	0.85 (0.80–0.91)	<0.0001	1.04 (0.98–1.11)	0.222
Dementia	0.13 (0.11–0.17)	<0.0001	0.37 (0.30–0.47)	<0.0001
Depression	0.83 (0.78–0.89)	<0.0001	0.92 (0.85–0.99)	0.022
Diabetes	0.88 (0.84–0.92)	<0.0001	0.97 (0.92–1.02)	0.262
Heart failure	0.44 (0.41–0.47)	<0.0001	0.72 (0.67–0.78)	<0.0001
Hypertension	0.84 (0.81–0.88)	< 0.0001	1.15 (1.10–1.20)	<0.0001
Liver Disease	0.81 (0.66–0.99)	0.035	1.25 (1.02–1.52)	0.030
Malignancy	0.61 (0.57–0.66)	<0.0001	0.94 (0.87–1.01)	0.079
Peripheral vascular disease	0.76 (0.70–0.82)	<0.0001	1.09 (1.00–1.19)	0.053
Prior myocardial infarction	0.88 (0.83–0.94)	<0.0001	1.19 (1.12–1.27)	<0.0001
Psychotic disorder	0.58 (0.50–0.67)	<0.0001	0.63 (0.54–0.73)	<0.0001
Rheumatic disease	0.58 (0.50–0.67)	<0.0001	0.97 (0.88–1.06)	0.441
Renal failure	0.61 (0.54–0.70)	<0.0001	0.96 (0.83–1.10)	0.542
Revascularization	2.47 (2.35–2.60)	<0.0001	1.56 (1.47–1.66)	<0.0001
ST-elevation MI	1.22 (1.17–1.27)	<0.0001	0.88 (0.84–0.92)	<0.0001
Early statin after MI		<0.0001		<0.0001
High-intensity	Reference	Reference	Reference	Reference
Moderate-intensity	0.53 (0.51–0.56)	<0.0001	0.66 (0.63–0.69)	<0.0001
Low-intensity	0.42 (0.36–0.49)	<0.0001	0.91 (0.77–1.08)	0.270
None	0.29 (0.27–0.31)	<0.0001	0.60 (0.55–0.65)	<0.0001

Abbreviations: MI, myocardial infarction; sHR = subdistribution hazard ratio; PCI, percutaneous coronary intervention; CABG, coronary artery bypass grafting.

The average proportion of patients using ezetimibe during follow-up was 6.8 %. Ezetimibe therapy was used by 5.8 % at 1 year, 7.7 % at 5 years, and 11.7 % at 10 years after MI (Fig. 2). The proportion of patients who had discontinued post-MI ezetimibe therapy increased from 25.4 % at 1 year to 41.1 % at 5 years and 43.6 % at 10 years (Fig. 2).

### 3.2 Ezetimibe and mortality

**Fig. 3:**
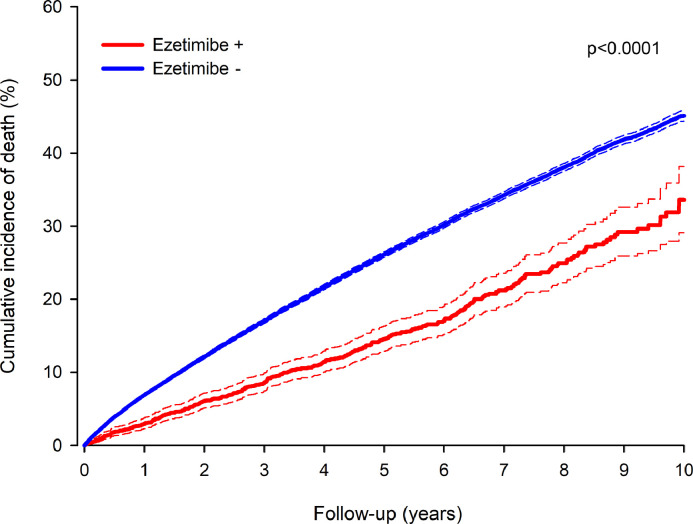
All-cause mortality after myocardial infarction by early usage of ezetimibe. Non-adjusted curves of 90-day MI survivors. Dashed lines represent 95 % confidence intervals.

A total of 16,787 patients died during the follow-up period. In the total study cohort, the 10-year all-cause mortality was 33.6 % in the patients with early ezetimibe use and 45.1 % in the patients without early ezetimibe use after MI (Fig. 3). The patients with early ezetimibe use had lower all-cause mortality after MI in a non-adjusted analysis (HR 0.57; CI 0.50–0.62; *P* < 0.0001) and after adjustment for age, sex, comorbidities, MI type, revascularization, statin usage and dose, and usage of other cardiovascular pharmacotherapies (adj.HR 0.77; CI 0.69–0.86; *P* < 0.0001). The E-value was 1.92 (CI 1.60–2.26).

Ezetimibe was associated with lower mortality in the subgroup analyses of men and women, different age-groups, patients with and without atrial fibrillation, diabetes, heart failure, or malignancy, revascularized and non-revascularized patients, and patients with and without early statin use after MI (Supplement Table 1). The association of early ezetimibe use with lower mortality was more pronounced in the patients not using statins (adj.HR 0.60; CI 0.48–0.76; *P* < 0.0001) than in those using statins (adj.HR 0.82; 0.73–0.92; *P* = 0.001), interaction *P* = 0.019. Ongoing ezetimibe therapy during follow-up was associated with lower mortality in a time-dependent analysis (adj.HR 0.53; CI 0.48–0.59; *P* < 0.0001).

Baseline cardiovascular comorbidities, increasing age, and lack of revascularization were associated with higher 10-year mortality in patients with early ezetimibe use after MI (Supplemental Table 2).

## 4 Discussion

This observational, nationwide cohort study investigated the use of ezetimibe and association with mortality after MI. Younger age was one of the strongest predictors of ezetimibe use. Ezetimibe was discontinued by 44 % of patients during the 10-year follow-up. Furthermore, ezetimibe use was independently associated with a lower risk of death after MI.

Ezetimibe acts by inhibiting the Niemann-Pick C1-Like 1 transporter protein, thereby reducing cholesterol absorption from the intestine. This results in the compensatory activation of HMG-CoA reductase and cholesterol synthesis, and thus a synergistic effect can be obtained when ezetimibe is used together with statin therapy. Ezetimibe lowers LDL levels by 19–23 % when added to statins [[16], [17], [18]]. Ezetimibe is indeed mainly used together with statins to attain lipid-lowering goals, but it is also indicated in situations where statins are not tolerated.

Data on ezetimibe use after MI is scarce. In the present study, we found that 6.8 % of patients initiated ezetimibe therapy by 1 year and 19.8 % by 10 years after MI. This compares to a previous US veteran study with a predominantly male study population that reported 5.7 % of patients receiving ezetimibe within 1 year after MI or elective revascularization during the same study period [19]. Notably, we found age to be one of the strongest predictors of ezetimibe use after MI, with patients aged < 60 years being 6.7-fold more likely to receive ezetimibe during follow-up than patients aged ≥ 80 years. This age discrepancy increased during follow-up, indicating less frequent or absent follow-ups and less aggressive secondary prevention in older patients. Aggressive LDL reduction is nevertheless also efficient in older MI patients [20], and ezetimibe is effective after ACS regardless of the patient's risk profile [21]. In line with a previous observation [22], we found women to be more likely to receive ezetimibe than men after MI when accounting for age and other covariables. Analyses were adjusted with initial statin intensity, which is found to be less intensive in women after MI [23].

The continuity of ezetimibe was poor in our study, with 44 % of patients with MI discontinuing therapy during follow-up. Notably, discontinuation of ezetimibe was previously found to be as high as 78 % by 3 years in all dyslipidemia patients [24] showing poorer adherence in primary prevention. Curiously, adherence to ezetimibe is poorer than adherence to statins, as 24 % of patients had discontinued statins by 10 years after MI in a previous study that used the same databases as the current study [11]. However, discontinuation of high-intensity statins is more common compared to lower-dose statins [25]. The recent Randomized Comparison of Efficacy and Safety of Lipid‐Lowering With Statin Monotherapy Versus Statin/Ezetimibe Combination for High‐Risk Cardiovascular Diseases (RACING) trial found discontinuation or therapy de-intensification to be less common with a moderate-intensity statin-ezetimibe combination than with high-intensity statins [26] while combination therapy lowered LDL levels more [27].

Ezetimibe is generally very well tolerated. Mild gastrointestinal symptoms and myopathy are commonly reported, but the latter may mainly be due to concomitant statin therapy. However, a lack of adherence to and persistence with lipid-lowering therapy is a problem regardless of the type of therapy [24,28]. It is unlikely that, in our population of MI patients, the de-escalation of lipid-lowering therapy or serious adverse effects would explain the major proportion of ezetimibe discontinuation. Thus, the implementation of patient and physician educational programs, pharmacy-based programs, and routine monitoring may be effective ways to improve ezetimibe adherence [29,30].

The IMPROVE-IT trial of 18,144 patients showed that the addition of ezetimibe to moderate-intensity statins reduced the combined end point of cardiovascular death, recurrent ACS, coronary revascularization, or stroke after ACS (HR 0.94; CI 0.89–0.99), but there was no difference in all-cause mortality [6]. A recent network meta-analysis of 7 randomized trials found that the addition of ezetimibe to statins reduced both major cardiovascular events (HR 0.83; CI 0.70–0.98) and all-cause death (OR 0.55; CI 0.34–0.89) in percutaneously treated patient with ACS [31]. However, real-world evidence on ezetimibe is scarce, with a recent observational study finding lower all-cause mortality in patients with early post-MI ezetimibe-statin combination therapy when compared to statin monotherapy [32].

We found lower all-cause mortality after MI in the patients with ezetimibe therapy. Ezetimibe was associated with lower mortality in both early ezetimibe users and in patients using ezetimibe during follow-up. Adding ezetimibe to the pharmacotherapy plan early after MI was associated with a lower risk of death regardless of age, sex, atrial fibrillation, diabetes, heart failure, malignancy, revascularization, or statin use or dose. Our results underline the importance of intensive LDL lowering in all patients with MI [1].

Previous studies advocate aggressive LDL lowering rather than stepwise treatment intensification after MI [26,32,33]. Aggressive upfront LDL-lowering therapy is supported by the facts that lipid levels are unfrequently controlled [34,35] and the intensity of LDL-lowering therapy remains largely unaltered after MI [11,19,35]. The most recent ACS guideline from the European Society of Cardiology gives a class I recommendation for adding ezetimibe to the highest tolerated statin intensity if the patient's LDL level is <1.4 mmol/L (<55 mg/dL) and a class IIb recommendation for initiating a ezetimibe-statin combination in statin-naïve patients and patients with low-intensity statins during MI admission [36].

### 4.1 Limitations

The current study has limitations. The main limitations are related to the retrospective design and available data. We used an all-comer, nationwide observational study design with combined national registries. The number of covariables were studied and adjusted for, yet residual confounding by non-recognized factors is possible and may have caused bias and influenced the results. The major limitations and potential causes of bias are lack of data on LDL levels and clinical follow-ups after MI. Moreover, we did not have access to more detailed clinical information on the patients, including laboratory measures, angiographical data, smoking status, or other lifestyle factors. However, the benefits of ezetimibe are not dependent on baseline LDL levels [37]. The E-value indicates that the observed adjusted HR of 0.77 in the long-term mortality for early ezetimibe users vs non-users could be explained by an unmeasured confounding associated with both ezetimibe use and death at a risk ratio of ≥ 1.9-fold each; however, weaker confounding could not accomplish this [15]. In addition to the intention-to-treat analysis, a time-dependent on-treatment analysis showed an association of ongoing ezetimibe and a lower risk of death. However, on-treatment analysis does not control for reasons of treatment discontinuation [38] or for differences in patient follow-up, resulting to potential over-estimation of true association between ezetimibe and mortality.

In conclusion, ezetimibe use after MI was associated with lower mortality across the spectrum of patients with MI. However, ezetimibe initiation was limited and strongly inversely tied to patient age. Moreover, ezetimibe discontinuation was common. These results underline the benefit of ezetimibe after MI and the need for focusing on more aggressive treatment initiation as well as the prevention of treatment discontinuation.

## CRediT authorship contribution statement

**Ville Kytö:** Writing – review & editing, Writing – original draft, Visualization, Validation, Supervision, Resources, Project administration, Methodology, Investigation, Funding acquisition, Formal analysis, Data curation, Conceptualization. **Aleksi Tornio:** Writing – review & editing, Writing – original draft, Visualization, Supervision, Methodology, Conceptualization.

## Declaration of competing interest

The authors declare that they have no known competing financial interests or personal relationships that could have appeared to influence the work reported in this paper.

## Appendix Supplementary materials

Image, application 1
