# Supplementary material for: Ezetimibe use and mortality after myocardial infarction: A nationwide cohort study
Source: Am J Prev Cardiol. 2024 Jun 23;19:100702. doi: 10.1016/j.ajpc.2024.100702 (PMC11278110; doi:10.1016/j.ajpc.2024.100702)
Supplement: Supplementary file 1 [file mmc1.pdf]

Supplement

Supplemental Methods

Study Data

The data of study patients were collected and combined from the following mandated-by-law, nationwide registries.

- Hospital admissions, outpatient visits to specialist medical care, and emergency room admissions: diagnoses (International Classification of Diagnosis / ICD-10) and operational codes (Nordic Classification of Surgical Procedures) from the CRHC registry. Data from Jan 1st 2004 to index event.
- Cancer diagnosis data from the Finnish Cancer Registry. Data from Jan 1st 2004 to index event.
- Medication purchases of studied medications (Anatomical Therapeutic Chemical / ATC-codes and purchase dates).
- Entitlements to special reimbursements for prescription medications including entitlement codes and associated ICD-10 diagnoses. Data from Jan 1st 1964 to index event.
- Mortality dates. Data from index event to Dec 31st 2020.

Co-morbidities were detected from the data of index MI admission and data available prior to index event. Revascularization was detected from the data of index admission.

Ongoing Ezetimibe Use

Ongoing ezetimibe use was studied in 90-day intervals during the follow-up. The number of patients with an ezetimibe purchase in each 90-day period was detected using the study database. Patients were allowed to start or stop ezetimibe by each 90-day period without limitations. The proportion of patients with ezetimibe therapy was calculated as: Number of ezetimibe purchasers in period / ((Number of patients at risk at the beginning of period + Number of patients at risk at the end of period)/2). The average proportion of patients using ezetimibe during the whole study period was calculated as: Number of patient periods with ezetimibe / Number of total patient periods at risk.

Discontinuation of Ezetimibe Use

The proportion of patients who discontinued post-MI ezetimibe therapy was studied in 90-day intervals during the follow-up. To account for package size differences and potential hospital admissions, patients were defined to have discontinued ezetimibe treatment after a second consecutive 90-day period without ezetimibe. Patients were allowed to start or stop ezetimibe by each 90-day period without limitations. Discontinuation was studied in patients alive at the end of each 90-day period.

Supplemental Table 1. Association of early ezetimibe use and all-cause mortality in subgroup analyses. Analyses were adjusted with patient features, comorbidities, revascularization, and other medications listed in Table 1.

	**Non-adjusted 10-year mortality**		**Non-adjusted**		**Adjusted**	
**Patient group**	**Ezetimibe +**	**Ezetimibe -**	**HR (95%CI)**	***P* Value**	**adj. HR (95%CI)**	***P* Value**
**All patients**	33.6%	45.1%	0.56 (0.50-0.62)	<0.0001	0.77 (0.69-0.86)	<0.0001
**Sex**				0.157*		0.955*
**Men**	30.4%	40.2%	0.60 (0.52-0.68)	<0.0001	0.77 (0.67-0.89)	0.0003
**Women**	40.0%	54.4%	0.51 (0.43-0.60)	<0.0001	0.77 (0.66-0.90)	0.001
**Age (years)**				0.239*		0.726*
**≥ 80**	86.3%	88.9%	0.67 (0.55-0.81)	<0.0001	0.67 (0.55-0.84)	0.0003
**70-79**	51.8%	53.3%	0.81 (0.68-0.95)	0.013	0.74 (0.62-0.87)	0.001
**60-69**	27.8%	29.1%	0.89 (0.73-1.09)	0.264	0.79 (0.65-0.93)	0.026
**18-59**	12.2%	13.6%	0.80 (0.57-1.11)	0.176	0.80 (0.58-1.10)	0.172
**Atrial fibrillation**				0.951*		0.223*
**Yes**	61.9%	73.0%	0.56 (0.46-0.69)	<0.0001	0.87 (0.70-1.07)	0.176
**No**	29.6%	40.2%	0.57 (0.50-0.64)	<0.0001	0.74 (0.66-0.84)	<0.0001
**Diabetes**				0.852*		0.879*
**Yes**	48.7%	59.4%	0.54 (0.46-0.63)	<0.0001	0.78 (0.66-0.92)	0.003
**No**	27.6%	40.1%	0.53 (0.46-0.61)	<0.0001	0.77 (0.67-0.88)	0.0001
**Heart Failure**				0.208*		0.232*
**Yes**	67.3%	78.4%	0.62 (0.53-0.74)	<0.0001	0.84 (0.70-0.99)	0.049
**No**	26.8%	37.1%	0.54 (0.48-0.62)	<0.0001	0.73 (0.64-0.83)	<0.0001
**Malignancy**				0.261*		0.304*
**Yes**	60.7%	66.7%	0.58 (0.52-0.65)	<0.0001	0.68 (0.53-0.88)	0.004
**No**	31.0%	41.8%	0.50 (0.40-0.64)	<0.0001	0.79 (0.71-0.89)	<0.0001
**Revascularization**				0.035*		0.850*
**Yes**	27.3%	33.5%	0.67 (0.58-0.77)	<0.0001	0.76 (0.66-0.88)	0.0002
**No**	51.1%	65.4%	0.53 (0.46-0.63)	<0.0001	0.78 (0.67-0.91)	0.002
**Statin after MI**				<0.0001*		0.019*
**Yes**	31.9%	40.2%	0.63 (0.56-0.70)	<0.0001	0.82 (0.73-0.92)	0.001
**No**	43.7%	72.7%	0.36 (0.29-0.46)	<0.0001	0.60 (0.48-0.76)	<0.0001

* Interaction P-value.

Supplemental Table 2. Association of baseline features with 10-year all-cause mortality in patients using ezetimibe early after MI.

	Univariable		Multivariable	
**Variable**	**HR (95%CI)**	***P* Value**	**adj.HR (95%CI)**	***P* Value**
**Age (years)**		<0.0001		<0.0001
**≥ 80**	Reference	Reference	Reference	Reference
**70-79**	0.44 (0.34-0.58)	<0.0001	0.76 (0.72-0.79)	<0.0001
**60-69**	0.21 (0.16-0.28)	<0.0001	0.48 (0.45-0.51)	<0.0001
**18-59**	0.08 (0.05-0.11)	<0.0001	0.15 (0.13-0.17)	<0.0001
**Female sex**	1.32 (1.07-1.63)	0.011	0.83 (0.66-1.04)	0.104
**Medical history**				
**Atrial fibrillation**	2.69 (2.11-3.43)	<0.0001	1.43 (1.01-1.87)	0.010
**Cerebrovascular disease**	2.26 (1.77-2.89)	<0.0001	1.13 (0.86-1.47)	0.380
**Chronic pulmonary disease**	1.83 (1.43-2.36)	<0.0001	1.40 (1.07-1.82)	0.014
**Dementia**	4.07 (2.29-7.22)	<0.0001	1.43 (0.71-2.88)	0.311
**Depression**	1.41 (1.04-1.91)	0.003	1.28 (0.92-1.78)	0.136
**Diabetes**	1.89 (1.53-2.33)	<0.0001	1.46 (1.16-1.85)	0.001
**Heart failure**	4.38 (3.52-5.43)	<0.0001	2.06 (1.62-2.62)	<0.0001
**Hypertension**	1.88 (1.50-2.36)	< 0.0001	1.01 (0.78-1.30)	0.965
**Liver Disease**	1.60 (0.79-3.21)	0.190	1.75 (0.80-3.84)	0.160
**Malignancy**	1.98 (1.51-2.59)	<0.0001	1.17 (0.86-1.58)	0.318
**Peripheral vascular disease**	3.28 (2.58-4.16)	<0.0001	1.56 (1.18-2.05)	0.002
**Prior myocardial infarction**	1.93 (1.53-2.43)	<0.0001	1.20 (0.94-1.54)	0.143
**Psychotic disorder**	1.19 (0.55-2.27)	0.758	1.07 (0.51-2.23)	0.868
**Rheumatic disease**	1.63 (1.15-2.29)	0.006	1.08 (0.73-1.61)	0.704
**Renal failure**	4.29 (3.07-6.00)	<0.0001	2.34 (1.60-3.41)	<0.0001
**Revascularization**	0.40 (0.32-0.49)	<0.0001	0.57 (0.45-0.72)	<0.0001
**ST-elevation MI**	0.55 (0.43-0.70)	<0.0001	1.09 (0.83-1.43)	0.550
**Early statin after MI**		<0.0001		0.974
**High-intensity**	Reference	Reference	Reference	Reference
**Moderate-intensity**	1.16 (0.91-1.49)	0.221	0.95 (0.74-1.23)	0.687
**Low-intensity**	1.43 (0.89-2.30)	0.138	0.97 (0.63-1.50)	0.887
**None**	2.00 (1.50-2.67)	<0.0001	1.00 (0.73-1.38)	0.982
